# Bone Marrow-Derived Endothelial Progenitor Cells Contribute to Monocrotaline-Induced Pulmonary Arterial Hypertension in Rats via Inhibition of Store-Operated Ca^2+^ Channels

**DOI:** 10.1155/2018/4892349

**Published:** 2018-09-18

**Authors:** Ran Miao, Jun Wan, Jie Liu, Jason X.-J. Yuan, Jing Wang, Wanmu Xie, Zhenguo Zhai, Chen Wang

**Affiliations:** ^1^Medical Research Center, Beijing Chao-Yang Hospital, Capital Medical University, Beijing 100020, China; ^2^Key Laboratory of Respiratory and Pulmonary Circulation Disorders, Institute of Respiratory Medicine, Beijing 100020, China; ^3^Center for Respiratory Diseases, Department of Pulmonary and Critical Care Medicine, China-Japan Friendship Hospital, National Clinical Research Center for Respiratory Diseases, National Pulmonary Embolism & Pulmonary Vascular Diseases Research Group, Beijing 100029, China; ^4^Department of Physiology, School of Basic Medicine, Capital Medical University, Beijing 100069, China; ^5^Division of Translational and Regenerative Medicine, The University of Arizona College of Medicine, Tucson, Arizona, USA

## Abstract

**Purpose:**

This study aimed to explore whether bone marrow- (BM-) derived endothelial progenitor cells (EPCs) contributing to monocrotaline- (MCT-) induced pulmonary arterial hypertension (PAH) in rats via modulating store-operated Ca^2+^ channels (SOC).

**Methods:**

Sprague Dawley (SD) rats were assigned into MCT group (n = 30) and control group (n = 20). Rats in MCT group were subcutaneously administered with 60 mg/kg MCT solution, and rats in control group were injected with equal amount of vehicle. After 3 weeks of treatment, right ventricular systolic pressure (RVSP) and right ventricular hypertrophy index (RVHI) of two groups were measured, and BM-derived EPCs were isolated. Immunochemistry identification and vasculogenesis detection of EPCs were then performed. [Ca^2+^]_cyt_ measurement was performed to detect store-operated calcium entry (SOCE) in two groups, followed by determination of Orai and canonical transient receptor potential (TRPC) channels expression.

**Results:**

After 3 weeks of treatment, there were significant increases in RVSP and RVHI in MCT group compared with control group, indicating that MCT successfully induced PAH in rats. Moreover, the SOCE ([Ca^2+^]_cyt_ rise) in BM-derived EPCs of MCT group was lower than that of control group. Furthermore, the expression levels of Orai3, TRPC1, TRPC3, and TRPC6 in BM-derived EPCs were decreased in MCT group in comparison with control group.

**Conclusions:**

The SOC activities were inhibited in BM-derived EPCs of MCT-treated rats. These results may be associated with the depressed expression of Orai3, TRPC1, TRPC3, and TRPC6, which are major mediators of SOC.

## 1. Introduction

Pulmonary arterial hypertension (PAH) is a fatal disorder characterized by an increase in pulmonary vascular resistance [1, 2]. It always leads to right ventricular (RV) failure and death [3, 4]. Despite advances in therapeutic options, this disease represents an incurable disease due to progressive clinical deterioration and an unacceptably high early mortality [5, 6]. Therefore, elucidation of key pathological mechanism underlying PAH development is still imperative.

Accumulating evidences have confirmed that excessive pulmonary vascular remodeling is responsible for the elevated pulmonary vascular resistance in PAH [7–9]. In pulmonary arterial smooth muscle cells (PASMCs), the rise in cytosolic free Ca^2+^ concentration ([Ca^2+^]_cyt_) is identified as a key trigger for promoting the proliferation of PASMCs and pulmonary vasoconstriction, both leading to pulmonary vascular remodeling [10–13]. Moreover, the profound pulmonary vascular remodeling and alterations in Ca^2+^ homeostasis in PASMCs may result in the development of PAH [14]. These findings support the pathogenic role of Ca^2+^ signaling in PAH.

Endothelial progenitor cells (EPCs) are considered to be important in maintaining vascular homeostasis, which can be mobilized from the bone marrow (BM) and resident locally in the lung [15]. EPCs are found to have a key role in the endothelial repair [16, 17]. It is reported that BM-derived EPCs can repair the monocrotaline- (MCT-) damaged lung in the rat MCT model of PAH [18]. Moreover, EPCs can induce neovascularization, suggesting the promising clinical application of the EPCs cell therapy to PAH [19]. However, the possible mechanism of EPCs in regulating pulmonary vascular remodeling during PAH development is largely unknown.

Notably, store-operated Ca^2+^ channels (SOC) is expressed in human EPCs [20]. Given the pathogenic role of Ca^2+^ signaling in PAH, the present study investigated whether BM-derived EPCs contributed to PAH in the MCT rat model via modulating SOC. To study this, we established the MCT rat model that was widely used to investigate PAH in rodents [20–22]. Then BM-derived EPCs were isolated. [Ca^2+^]_cyt_ measurement was performed to detect store-operated calcium entry (SOCE) in BM-derived EPCs of MCT rat model and controls, followed by determination of SOC regulators, Orai, and canonical transient receptor potential channel (TRPC) expression. Our findings will provide a new insight for better understanding of PAH pathogenesis.

## 2. Materials and Methods

### 2.1. Animals and Treatment

A total of 50 male Sprague Dawley (SD) rats (weighing 150-180 g) were obtained from Beijing Vital River Laboratory Animal Technology Co., Ltd. (Beijing, China). Rats were divided into MCT group (n = 30) and control group (n = 20). Rats in MCT group were subcutaneously administered with 60 mg/kg MCT solution 25 mg/ml diluted in vehicle (1:4 mixture of dehydrated ethanol-normal saline). Rats in control group were injected with equal amount of vehicle. This study was approved by the institutional ethical committee for animal care and use. During the treatment period, the behavior and general status were observed daily.

### 2.2. Measurement of Pulmonary Hemodynamic and Right Ventricular Hypertrophy

After 3 weeks of treatment, the rats were intraperitoneally anesthetized with 35mg/kg pentobarbital sodium (Abbott Laboratories, Montreal, Canada). Right ventricular systolic pressure (RVSP) of rats in each group was measured by inserting a Millar catheter (Millar, Inc., TX, USA) into RV. Moreover, the RV was separated from the left ventricle (LV) and septum (S) for further detection of RV hypertrophy. The right heart hypertrophy index (RVHI) [RV/(LV+S)] was calculated as the ratio of RV weight to (LV+S) weight.

### 2.3. Isolation of Rat BM-Derived EPCs

Rats in each group were sacrificed by exsanguination, and BM was then aspirated from bilateral femurs and tibias of rats aseptically. Using density gradient centrifugation Histopaque®-1083 solution (Sigma-Aldrich, MS, USA), mononuclear cells (MNCs) were isolated from BM. To produce EPCs, BM-isolated MNCs were then resuspended in EGM-2 MV medium (Lonza, MD, USA), seeded into fibronectin (5 *μ*g/cm^2^) coated six-well plates at a density of 3 × 10^6^/cm^2^ and maintained at a 37°C incubator for 8 days. EGM-2 MV medium was replaced every two days.

### 2.4. Immunochemistry Identification

After 8 days of incubation, the fibronectin-adherent EPCs were identified by incubation with 10 *μ*g/ml of fluorescently labeled acetylated-low-density lipoprotein (Dil-ac-LDL; Molecular Probes, Eugene, OR, USA) overnight and 10 *μ*g/ml of fluorescently FITC-labeled* Ulex europaeus* agglutinin 1 (UEA-1; Sigma-Aldrich, MS, USA) for 4 h at room temperature using an immunochemistry method [4, 23]. The images were captured by Leica-SP5 confocal microscopy (Leica, Germany), and both FITC-UEA-1 and Dil-ac-LDL positive cells were considered as EPCs.

### 2.5. Detection of Vasculogenesis

To mimic vasculogenesis of EPCs, the vascular network formation was observed. Briefly, 24-well plates were presolidified Matrigel (BD Biosciences, MA, USA) for 30 minutes. EPCs were seeded into 24-well plates containing 500 *μ*l EGM-2-MV medium at a density of 7.5 × 10^4^ cells/2cm^2^ Matrigel. After incubation for 5 h, the developing vascular network in 10 fields was observed under a microscope (Nikon, Japan). The length of vascular network per field was calculated.

### 2.6. [Ca^2+^]_cyt_ Measurement

According to the protocols described previously [24, 25], [Ca^2+^]_cyt_, defined as the ratio of fluorescence intensities of 340 to 380 nm wavelengths (F340/F380), was monitored using fura-2 acetoxymethyl ester (Invitrogen-Molecular Probes, Eugene, OR) and then imaged with NIS Elements 3.2 software (Nikon).

To determine whether the different amplitude of [Ca^2+^]_cyt_ increase between MCT and control groups was caused by SOCE, 10 *μ*M CPA was extracellularly applied, which is a sarco/endoplasmic reticulum Ca^2+^-ATPase inhibitor that induces Ca^2+^ influx. The [Ca^2+^]_cyt_ rise (ΔRatio) of MCT and control groups was then detected.

### 2.7. Real-Time PCR

Total RNA was extracted from EPCs in MCT and control groups using TRIzol® reagent (Invitrogen, Burlington, ON, Canada). The quality and concentration of total RNA were then determined with a spectrophotometer (NanoDrop 2000, Thermo Scientific, USA). Reverse transcription into cDNA was conducted using the PrimeScriptTM RT Master Mix Kit (Takara, Japan). The expression levels of Orai and TRPC channel were then detected by real-time PCR on the Applied Biosystems Real-Time PCR System 7500 Fast (Applied Biosystems, Foster City, CA, USA). The primers (forward and reverse, 5'- 3') for amplification of genes were as follows: Orai 1: AAGTTCTTACCGCTCAAGAGGCAG and AGCGGTAGAAGTGAACGGCAAAGA; Orai 2: TGTGGGTCTCATCTTCGTGGTCTT and TGAGCTTGTGCAGTTCCTCGATCT; Orai 3: TAGTGCCTGCACCACTGTGTTAGT and ATTGTGGATGTTGCTCACGGCTTC; TRPC7: AGACACGGAAGAGGTGGAAGCAAT and AGTTAGGGTGGGCAACGAACTTCT; TRPC6: TCTGGCTGCTCATTGCCAGGAATA and AGAGTGGCTGAAGGAGTCATGCTT; TRPC5: AGTTCACACCAGACATCACACCCA and TGAACTGGACACACACTCCACACA; TRPC4: AGTTTATCTGCCACACAGCCTCCT and AGTCCGCCATCCCACATCTGTTTA; TRPC3: TCTTCCTGGGTCTGCTTGTGTTCA and TGTCCATGTGAACTGGGTGGTCTT; TRPC2: TCCTGTGAAGATCAGCCATGTGGT and TGTCTGGGTTCAGCAAGTTCTCCA; and TRPC1: ACAGAAGATGCAGAGCACAGACCA and AAGTCCGAAAGCCAAGCAAATCCC. Each sample was analyzed in triplicate. Cycling parameters were set as follows: 50°C for 2 min and 95°C for 10 min, followed by 40 cycles of 95°C for 15 s, 60°C for 30 s, and 72°C for 30 s.

### 2.8. Statistical Analysis

All experiments were independently repeated three times. All measurement data were presented as the mean ± standard deviation (SD) and analyzed for significant difference by using an unpaired Student's *t*-test Prism 5 software (GraphPad Software, Inc., La Jolla, CA, USA). A value of P < 0.05 indicated a statistically significant result.

## 3. Results

### 3.1. The General Status of Animals

During the animal experiment, 2 and 3 rats in MCT group died in the third and fourth weeks after MCT injection, respectively. As a result, 45 rats were enrolled in this study. After 3 weeks of treatment, rats in MCT group appeared to obviously have asarcia, dyspnea, and chest and ascites formation, together with liver congestion and swelling, heart enlargement, right ventricular hypertrophy, and other heart failure manifestations. Rats in control group did not exhibit any abnormities.

### 3.2. MCT-Induced PAH in Rats

**Figure 1:**
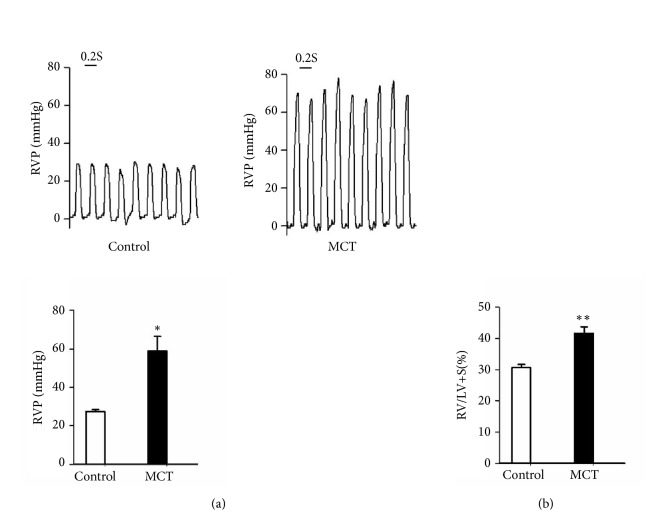
Monocrotaline (MCT) induced pulmonary arterial hypertension (PAH) in rats after 4 weeks of MCT treatment. (a) RVSP of rats in each group. (b) The RVHI of rats in each group. RVSP: right ventricular systolic pressure; RVHI: right ventricular hypertrophy index. *∗* P < 0.05 and *∗∗* P < 0.01 compared with control group.

MCT was used to induce PAH in rats in this study. The results showed that rats in MCT group developed PAH after 3 weeks of MCT treatment as reflected by a remarkable increase in RVSP: 59.40 ± 8.13 mmHg in MCT rats versus 27.45 ± 0.89 mmHg in control rats (P< 0.05, Figure 1(a)). Moreover, the RVHI in MCT group (41.69 ± 2.00%) was significantly increased compared with that in the control group (31.00 ± 1.00%) (P< 0.01, Figure 1(b)). These data indicated that MCT successfully induced PAH in rats.

### 3.3. Identification of BM-Derived EPCs

**Figure 2:**
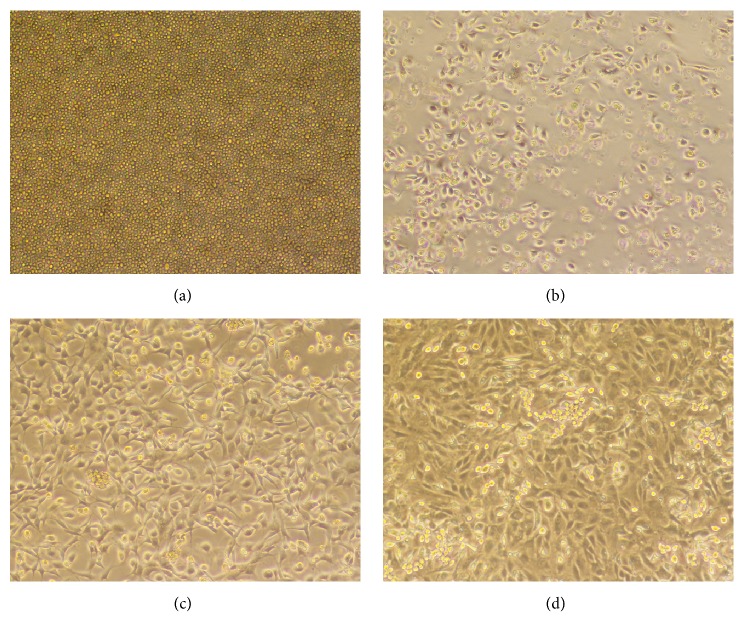
The morphological changes of bone marrow- (BM-) derived endothelial progenitor cells (EPCs) after culture for 1 (a), 4 (b), 6 (c), and 8 (d) days in the selective medium.

The morphological changes of EPCs were observed after culture for 1, 4, 6, and 8 days in the selective medium. After 1 day of culture, cells were adhered to the wall, but their morphology was not uniform and most of these cells were round (Figure 2(a)); some cells were polygonal or spindle-shaped after 4 days (Figure 2(b)); the majority of cells were polygonal or spindle-shaped after 6 days (Figure 2(c)); spindle-shaped or polygonal cells showed dominant growth after 8 days, paving stone-like arrangement and clear cell gaps (Figure 2(d)).

**Figure 3:**
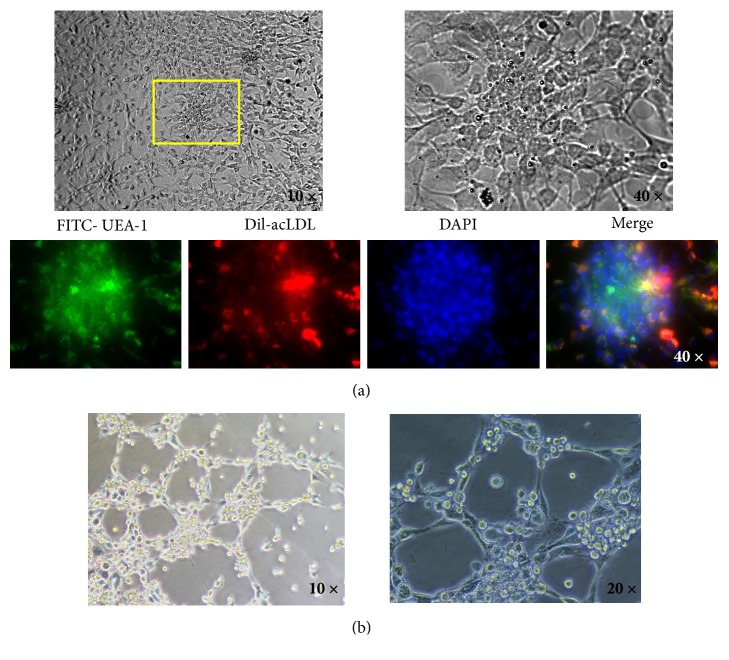
BM-derived EPCs identification with immunochemistry (a), as well as the vascular network formation test (b).

Furthermore, immunochemistry identification was performed after culture for 8 days in the selective medium. Double stained with FITC-UEA-1 and Dil-ac-LDL, EPCs in the adherent MNCs were identified (Figure 3(a)). Moreover, the results showed that 84.40 ± 8.06% of adherent MNCs were identified as double-positive EPCs, confirming that highly purified BM-derived EPCs were successfully isolated.

### 3.4. Functional Analysis of BM-Derived EPCs by Detection of Vasculogenesis

To further confirm the function of BM-derived EPCs, the vasculogenic potential of EPCs was detected by the vascular network formation test. EPCs were seeded onto the Matrigel for incubation for 5 h. The results showed that the average length of vascular network per field of view was 9.78 ± 0.67 mm (Figure 3(b)).

### 3.5. MCT Decreased CPA-Induced SOCE in BM-Derived EPCs

**Figure 4:**
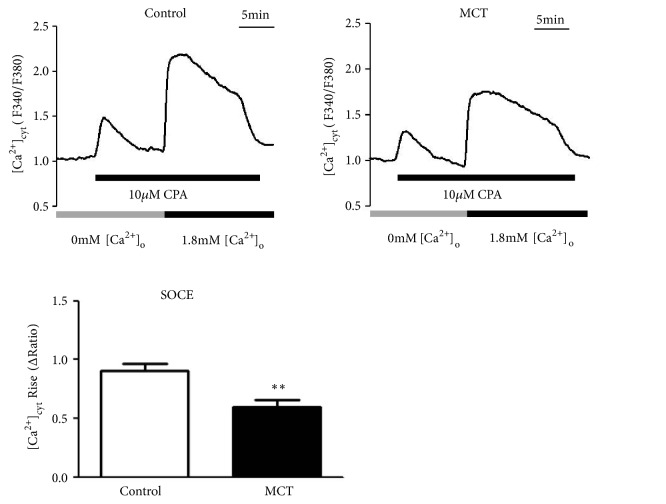
The [Ca^2+^]_cyt_ rise (SOCE) of MCT and control group. *∗∗* P < 0.01 compared with control group.

To determine whether MCT could regulate SOC in BM-derived EPCs, 10 *μ*M CPA was extracellularly applied to detect the effects of MCT on SOCE. As shown in Figure 4, the [Ca^2+^]_cyt_ rise (ΔRatio) of MCT group (0.60±0.21) was significantly lower than that in control group (0.91±0.23) (P< 0.01), indicating that MCT decreased CPA-induced SOCE.

### 3.6. The Effects of MCT on the Expression of Orai and TRPC Channel

**Figure 5:**
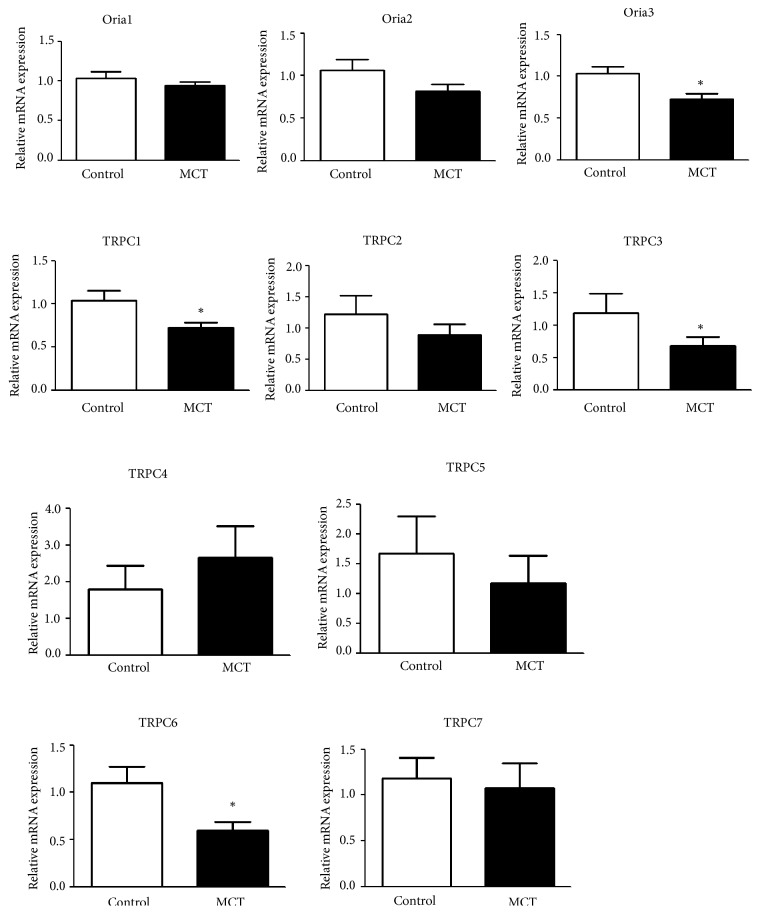
The expression of Orai and TRPC channels, including Orai1, Orai2, Orai3, TRPC1, TRPC2, TRPC3, TRPC4, TRPC5, TRPC6, and TRPC7, in MCT and control group. *∗* P < 0.05 compared with control group.

To further investigate the regulatory mechanism of MCT on SOC, we detected the Orai and TRPC channel expressions, including Orai1-3 and TRPC1-7. In comparison with control group, the expression levels of Orai3, TRPC1, TRPC3, and TRPC6 in BM-derived EPCs were significantly downregulated in MCT group (all P < 0.05, Figure 5), indicating that MCT decreased SOCE possible via decreasing the expression of these channel molecules.

## 4. Discussion

PAH is a degenerating and devastating disease with limited treatment options [26]. Elucidation of the key mechanism underlying PAH will facilitate the development of effective therapeutic strategy for this disease. In this study, the MCT rat model of PAH was successfully established as reflected by a remarkable increase in RVSP and RVHI. Moreover, the delightful results were obtained that the SOCE ([Ca^2+^]_cyt_) rise in BM-derived EPCs of MCT rat model was significantly inhibited. Furthermore, the expression levels of Orai3, TRPC1, TRPC3, and TRPC6 were markedly decreased in BM-derived EPCs of MCT rat model. These data imply that BM-derived EPCs could be involved in MCT-induced PAH in rats via inhibiting SOCE and related channel expression.

Intracellular Ca^2+^ signaling, as an important second messenger for cell proliferation, is found to play an important role in numerous physiological and pathophysiological processes in PASMCs, like proliferation and hypertrophy [27]. SOCE is a ubiquitous Ca^2+^ entry pathway that is involved in the control of various physiological functions in various cell types [28]. When intracellular Ca^2+^ stores are depleted, SOC can mediate Ca^2+^ influx and increase [Ca^2+^]_cyt_ [29]. Moreover, TRPC-dependent SOC is confirmed as an important pathway to mediate the development of MCT-induced PAH [14]. Lin et al. revealed that the enhanced SOCE is responsible for the chronic hypoxia-induced pulmonary hypertension in rats [30]. Zhou et al. demonstrated that SOC regulated endothelial hyperpermeability in severe PAH [31]. These data confirm the pathological role of SOC in PASMCs during PAH development. However, SOC may play a dual role in different cell types. A previous study has shown that SOCE is an important factor in regulating the functions of EPCs and the SOCE inhibition reduces the proliferation and migration of EPCs during atherosclerosis [32]. Wang et al. indicated that SOC inhibition could prevent H_2_O_2_-induced apoptosis, thus exerting a protective effect on EPCs [33]. Lodola et al. demonstrated that SOC was remodeled and subsequently regulated in vitro angiogenesis in EPCs isolated from tumoral patients [34]. In our study, SOC was inhibited by MCT in BM-derived EPCs of MCT-treated rats. Given the key role of SOC in EPCs, we speculate that BM-derived EPCs may prevent MCT-induced PAH in rats possible via activation of SOC.

Furthermore, both Orai and TRPC proteins are proposed to form SOC [35]. Increasing evidence has suggested the important roles of Orai and TRPC channels in PAH. Orai1, Orai12, and Orai3 can promote SOCE in PASMCs and may serve as potential therapeutic targets for chronic hypoxia-induced pulmonary hypertension [36]. Dragoni et al. suggested that Orai3 is overexpressed in primary myelofibrosis-endothelial colony forming cells (ECFCs) that are EPC subset and thus resulted in the upregulation of SOCE [37]. In this study, Orai3 expression was markedly decreased in BM-derived EPCs of MCT rat model. Considering the key role SOC in PAH, we speculate that inhibition of SOCE due to the downregulation of Orai3 in BM-derived EPCs may play a key role in MCT-induced PAH. In addition, TRPC1 is a major constituent of SOCE and overexpression of TRPC1 can promote SOCE-induced vasoconstriction in rat pulmonary artery [38]. TRPC1 deficiency is found to impair the functions of EPCs on regulating angiogenesis [39]. Moreover, TRPC3 channels are found to be involved in the development of hypertension and its related complications [40]. Poteser et al. indicated that TRPC3 could regulate Ca^2+^ signaling in somatic EPCs [41]. TRPC3-mediated Ca^2+^ signaling in ECFCs is developed as a promising strategy for improving therapeutic angiogenesis in failing hearts [42]. Furthermore, TRPC6 is shown to be critically involved in the disease states of pulmonary vasculature [43]. Yu et al. revealed that a unique genetic variant of the TRPC6 gene promoter might result in pulmonary vascular abnormality in idiopathic PAH by linking abnormal TRPC6 transcription to nuclear factor-*κ*B activity [44]. Additionally, functional interaction between TRPC1 and TRPC6 can mediate Ca^2+^ entry in endothelial cells to promote lung vascular permeability [45]. Blockade of TRPC3 and TRPC6 could be a promising therapeutic strategy for PAH treatment [46]. In this study, the expression of the store-operated TRPC1, TRPC3, and TRPC6 channels was decreased in BM-derived EPCs of MCT rat model, suggesting that BM-derived EPCs may be implicated in MCT-induced PAH via decreasing the expression of these channel molecules.

In conclusion, The SOC activities were inhibited in BM-derived EPCs of MCT-treated rats. These results may be associated with and the depressed expression of Orai3, TRPC1, TRPC3, and TRPC6, which are major mediators of SOC. Our findings may provide a physiological basis for the potential clinical application of the EPCs cell therapy to PAH.

## Data Availability

The data used to support the findings of this study are included within the article.

## Additional Points

*Highlights*. (1) MCT-induced PAH in rats successfully. (2) SOCE was decreased by MCT in BM-derived EPCs of MCT-treated rats. (3) The expressions of Orai3 and TRPC1, 3 and 6, were decreased in EPCs of MCT rats.
